# Analytical Modeling of Flowrate and Its Maxima in Electrochemical Bioelectronics with Drug Delivery Capabilities

**DOI:** 10.34133/2022/9805932

**Published:** 2022-03-04

**Authors:** Raudel Avila, Yixin Wu, Rinaldo Garziera, John A. Rogers, Yonggang Huang

**Affiliations:** ^1^Department of Mechanical Engineering, Northwestern University, Evanston, IL 60208, USA; ^2^Department of Materials Science and Engineering, Northwestern University, Evanston, IL 60208, USA; ^3^Querrey Simpson Institute for Bioelectronics, Northwestern University, Evanston, IL 60208, USA; ^4^Dipartimento di Ingegneria ed Architettura, Università di Parma, Italy; ^5^Department of Biomedical Engineering, Northwestern University, Evanston, IL 60208, USA; ^6^Department of Electrical and Computer Engineering, Northwestern University, Evanston, IL 60208, USA; ^7^Department of Chemistry, Northwestern University, Evanston, IL 60208, USA; ^8^Department of Neurological Surgery, Northwestern University, Feinberg School of Medicine, Chicago, IL 60611, USA; ^9^Department of Civil and Environmental Engineering, Northwestern University, Evanston, IL 60208, USA

## Abstract

Flowrate control in flexible bioelectronics with targeted drug delivery capabilities is essential to ensure timely and safe delivery. For neuroscience and pharmacogenetics studies in small animals, these flexible bioelectronic systems can be tailored to deliver small drug volumes on a controlled fashion without damaging surrounding tissues from stresses induced by excessively high flowrates. The drug delivery process is realized by an electrochemical reaction that pressurizes the internal bioelectronic chambers to deform a flexible polymer membrane that pumps the drug through a network of microchannels implanted in the small animal. The flowrate temporal profile and global maximum are governed and can be modeled by the ideal gas law. Here, we obtain an analytical solution that groups the relevant mechanical, fluidic, environmental, and electrochemical terms involved in the drug delivery process into a set of three nondimensional parameters. The unique combinations of these three nondimensional parameters (related to the initial pressure, initial gas volume, and microfluidic resistance) can be used to model the flowrate and scale up the flexible bioelectronic design for experiments in medium and large animal models. The analytical solution is divided into (1) a fast variable that controls the maximum flowrate and (2) a slow variable that models the temporal profile. Together, the two variables detail the complete drug delivery process and control using the three nondimensional parameters. Comparison of the analytical model with alternative numerical models shows excellent agreement and validates the analytic modeling approach. These findings serve as a theoretical framework to design and optimize future flexible bioelectronic systems used in biomedical research, or related medical fields, and analytically control the flowrate and its global maximum for successful drug delivery.

## 1. Introduction

Controlled and targeted drug delivery of pharmacological agents in organs/tissues has helped researchers study local biological responses to drug treatments and determine the drug efficacy while mitigating unwanted side effects often present in systemic drug delivery strategies [1, 2]. The approaches and technologies in drug delivery vary significantly depending on factors related to the type of drug and anatomical target location. A review of drug delivery technologies presented in [3, 4] discusses the evolution of drug delivery approaches that range from tailored nanoparticles that drive the active drug agents through biological membranes to implantable microsystem with injectable probes that enable targeted drug delivery in organs/tissues of interest.

Examples of injectable drug delivery systems include intracerebral [5, 6] and intra-arterial [7] injections in the brain, kidneys, eyes, ears, and lymphatic system of small- (mice) and medium-sized (cats) animals that are aimed at enhancing the drug effectiveness when treating affected areas. For a timely and successful drug delivery, the flowrate must be accurately controlled as excessively high flowrates can exert dangerous stresses on fragile surrounding tissue [8, 9] and excessively low flowrates can obstruct the fluidic probes and result in an incomplete/ineffective delivery [10].  Table 1 shows a list of reported flowrate ranges used in drug delivery applications that vary depending on the size of the animal and target location. In reported injectable drug delivery systems for small animals, the drug flowrate can vary in the range of tens of nanoliters per minute to hundreds of microliters per minute depending on the drug dose and intended application such as behavioral neuroscience [10, 11] or cancer therapeutics [12–14]. For this reason, accurately controlling the flowrate and its maximum value during the drug delivery process is important to ensure safe delivery of the drug without imposing stresses that can damage the surrounding soft tissues in small animals.

The rapid development of microtechnology in biomedical research has enabled wireless flexible bioelectronics with drug delivery capabilities in miniaturized form factors that integrate chemical, mechanical, and fluidic interfaces to manipulate small fluid volumes in a programmable fashion and realize targeted drug delivery in regions like the brain [7, 9, 10, 15, 16], peripheral nerves [11], and eyes [17]. To circumvent previous tethered delivery strategies that restrict animal motion, flexible bioelectronics utilize a wireless link that harvests energy from nearby electromagnetic sources and powers the corresponding subsystems to deliver the drug in freely moving animals while maintaining negligible device and thermal loads that can affect the animal behavior or alter the chemistry of the drug. For neuroscience research specifically, this aspect is especially important because minimizing the device load on freely moving animal allows to study the drug effects without any interference from the drug delivery actuation mechanisms [18–20].

**Figure 1:**
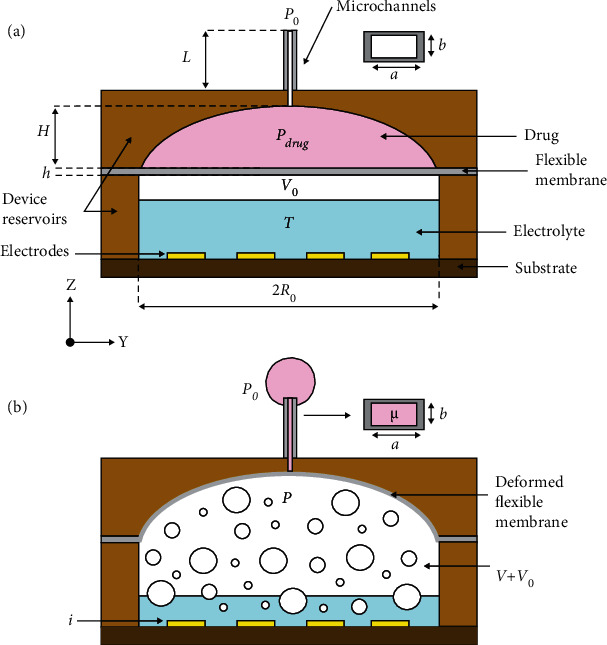
Simplified schematic of a bioelectronic layout used for drug delivery. (a) Before the drug delivery process, the electrolyte reservoir is partially filled where an initial volume of gas and the drug sits on top of the flexible membrane. (b) The gas formation process deforms the flexible membrane to pump the drug from inside the device through the microchannels and into the target location. The parameters involved in the drug delivery process are labeled through the schematic in their respective locations except the Young modulus, Poisson ratio, and stress-strain relationship of the flexible membrane.

Figure 1 shows a cross-sectional schematic of a typical flexible bioelectronic device used for drug delivery where all the geometric, environmental, fluidic, and mechanic parameters are labeled. The main components of the flexible bioelectronic include a set of interdigitated electrodes, an electrolyte chamber, a flexible membrane, a drug chamber, and microfluidic channels partially implanted in organs/tissues. To deliver the drug, electrical current flows through the electrodes—in direct contact with the electrolyte—to initiate an electrochemical reaction known as water hydrolysis. The byproducts of this electrochemical reaction are hydrogen and oxygen gas that accumulates in the electrolyte chamber and pressurizes the bottom side of a flexible membrane. In response to the accumulated gas pressure, the flexible membrane deforms into the form of a spherical cap and gradually pumps the drug, sitting on the top side of the flexible membrane, through a network of microfluidic channels which outlet is the target organ/tissue. By using electrochemistry, the drug can be pumped from inside the devices in a controlled fashion without generating excessive heat [11, 21, 22] or requiring external moving parts that can increase the complexity of injectable devices operating partially inside the tissue/organs of animals.

Several analytic models that combine more than 12 parameters involved in the drug delivery process related to geometric, environmental, fluidic, electrical, and flexible membrane mechanics into unique combinations of 3 nondimensional parameters related to the initial environmental pressure, initial gas volume, and the microfluidic resistance have been proposed [23]. These 3 nondimensional parameters group all the parameters involved in the drug delivery process and must be carefully selected to control the total delivery time and volume for accelerated drug delivery [24]. However, prior analytical models [23–25] focus on the total volume delivered over time for applications requiring accelerated delivery (e.g., life-saving medication) and do not satisfy the zero-flowrate initial condition which affects the flowrate over time, particularly the maximum flowrate. For injectable drug delivery systems targeting fragile tissues/organs in small animals, the maximum flowrate always occurs near the beginning of the drug delivery process and is the relevant quantity to control and adhere with reported experimental flowrate guidelines for administering certain types of drugs in experiments like the ones listed in Table 1 and to ensure that the drug is delivered safely without inducing excessive stresses that can damage the surrounding tissue/organs.

Here, we propose an analytical model that separates the drug delivery process into a “slow” variable that satisfies the zero-volume, but not zero-flowrate initial conditions, as used previously in [24, 25] to control the drug delivery time and volume, and a new “fast” variable that corrects the slow variable solution to satisfy both the zero-volume and zero-flowrate initial conditions and is used to control the flowrate and its maximum value without affecting the drug delivery time and volume. The new analytical model featuring a “fast” variable for flowrate control and an explicit formula for the maximum flowrate is validated by showing excellent agreement with the numerical results without using the “slow” and “fast” variables and serves as the theoretical framework to design the flexible bioelectronic devices with flowrate control capabilities that can be adjusted by unique combination of the nondimensional parameters depending on the target organ and drug delivery timeframe.

## 2. Results

### 2.1. Flexible Membrane Mechanics

Pumping the drug from inside the flexible device into the target location is achieved by inducing a pressure differential *P* − *P*_drug_ between the bottom and top surfaces of a flexible membrane which causes it to deform into the shape of the drug reservoir (e.g., spherical cap) with a maximum vertical displacement *H*. This deformation is governed by the flexible membrane geometrical (i.e., thickness *h* and radius *R*_0_) and mechanical properties (i.e., Young's modulus *E* and Poisson ratio *v* or alternatively a generalized hyperelastic strain energy density function), and the mechanics of deformation can be modeled according to the function *f*(*V*) that gives the relationship between pressure differential *P* − *P*_drug_ = *f*(*V*) applied to the flexible membrane and the volume *V* it expands.

During the delivery process, the flexible membrane transitions from bending-dominated (i.e., small displacement *H* ≪ *h*) to stretching-dominated deformation (i.e., large displacement *H* ≫ *h*) as it goes from flat into the shape of a spherical cap. Based on experiments for a bioelectronic device that targets the peripheral nerves [11] and numerical models of the drug delivery process [10, 23], the maximum flowrate always occurs near the beginning of the delivery process when the deformation is small, i.e., bending-dominated.

**Figure 2:**
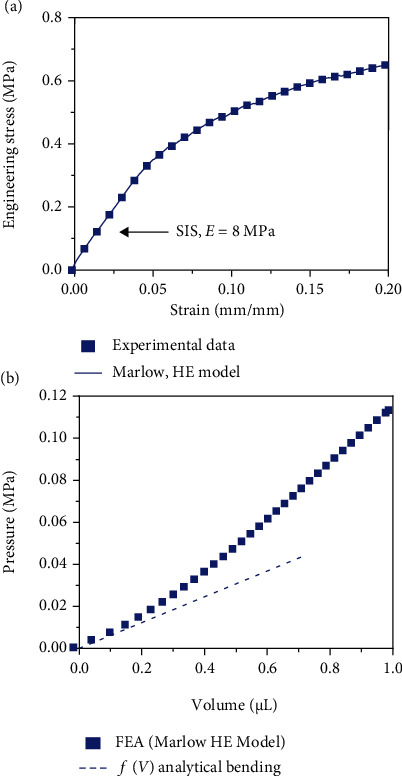
Mechanics of the flexible membrane. (a) Stress-strain experimental data for a representative SIS polymer selected for the flexible membrane (squares) and the Marlow hyperelastic (HE) model fit of the data (solid line). (b) Pressure-volume relationship for the SIS polymer obtained from FEA using the Marlow HE model (squares) and the pressure-volume relationship derived from plate theory for bending-dominated deformation (dashed line). The flexible membrane dimensions are thickness *h* = 150 *μm* and radius *R*_0_ = 1.20 mm.

For these bioelectronics with drug delivery capabilities, block copolymers like styrene-isoprene-styrene (SIS) have been used as flexible membrane for their soft mechanics and gas permeability properties which allow larger deformations and prevent the gas migration from the electrolyte chamber to the drug chamber [10]. Ideally, the stress-strain relationship of the block copolymers should be obtained from experimental testing (e.g., uniaxial and biaxial), when possible, to accurately model the flexible membrane expansion [32, 33].  Figure 2(a) shows the constitutive stress-strain relationship, obtained from uniaxial testing, of a SIS block copolymer sample. Young's modulus is calculated from the small strain (linear-elastic regime) as *E* = 8 MPa. For a representative bioelectronic device (i.e., thickness *h* = 150 *μ*m and radius *R*_0_ = 1.2 mm) previously used for drug delivery in the mouse brain [10], the function *f*(*V*) is obtained numerically using finite element analysis (FEA) as shown in Figure 2(b), which considers both bending and stretching effects and uses the Marlow hyperelastic model [33] to build the strain energy density function of the SIS block copolymer based on the stress-strain data shown in Figure 2(a). When the deformation of the flexible membrane is small (i.e., *H* ≪ *h*) such as near the beginning of the drug delivery process when the maximum flowrate occurs, a bending-dominated function *f*(*V*) is derived from plate theory [34] as

(1) $$\begin{array}{c} f\left(V\right)=P-P_{\text{drug}}=\frac{16Eh^{3}}{\pi R_{0}^{6}\left(1-v^{2}\right)}V\;\text{for }H\ll h, \end{array}$$

which establishes a linear relationship between pressure differential *P* − *P*_drug_ and the deformed volume *V* as shown in our previous work [24, 25] and has excellent agreement with the function *f*(*V*) obtained from FEA as shown in Figure 2(b) when the deformation of the flexible membrane is small. Generally, the function *f*(*V*) can be written nondimensionally as *G*(*V*^∗^) = (*R*_0_/(*Eh*))*f*(*V*^∗^*R*_0_^3^), where *V*^∗^ = *V*/*R*_0_^3^ is the nondimensional volume, which gives

(2) $$\begin{array}{c} G\left(V^{*}\right)=\frac{16h^{2}}{\pi R_{0}^{2}\left(1-v^{2}\right)}V^{*}, \end{array}$$

for bending-dominated deformation only. It gives a constant derivative with respect to *V*^∗^, and for an incompressible membrane material (i.e., *v* = 0.5),

(3) $$\begin{array}{c} G^{\prime }\left(V^{*}\right)=G^{\prime }\left(0\right)=\frac{16h^{2}}{\pi R_{0}^{2}\left(1-v^{2}\right)}=\frac{64}{3\pi }\frac{h^{2}}{R_{0}^{2}}, \end{array}$$

which will be used in subsequent sections to analyze the maximum flowrate.

Figure 2(b) shows that once the deformation of the flexible membrane becomes large or *H* ≫ *h*, the bending-dominated solution does not agree well with FEA as the stretching (and nonlinear deformation) effects in the membrane become relevant. However, for the analysis in this paper, the focus is the maximum flowrate which occurs when the deformation is small as explained in the Supplementary Information (Note 1), and thus, bending-dominated, not the volume or flowrate temporal response, requires the numerical and analytical function *f*(*V*) to agree over the entire pressure-volume range as shown in our previous work on stretching-dominated deformation [24, 25]. This validates the analytical bending-dominated function *f*(*V*) in Equation (1) instead of the FEA solution to control only the maximum flowrate in the drug delivery process.

### 2.2. Governing Equations of Drug Delivery

A typical wireless bioelectronic device features three main components: (1) electrochemical reservoir, (2) flexible membrane, and (3) microfluidic channels where each contributes to the drug delivery dynamics and affects the delivery time, flowrate, and its maxima and must be accounted for explicitly. The physics of wireless bioelectronics relying on electrochemistry as actuation method can be modeled approximately by the ideal gas law:

(4) $$\begin{array}{c} P\left(V+V_{0}\right)=nRT, \end{array}$$

where *P* is the pressure, *V* is volume change inside the electrolyte reservoir, *V*_0_ is the initial volume of gas in the electrolyte reservoir, *R* is the ideal gas constant (8.3144 J mol^−1^ K^−1^), *T* is the temperature of the electrolyte, and *n* is the number of moles, which is linear to the electrical current *i* in the electrodes and is given by *n* = ((3*i*)/(4*F*))*t* + *n*_0_ following Nernst equation [35]. Here, *t* is time, *F* is Faraday's constant (96485 C mol^−1^), the ratio 3/4 corresponds to water and must be changed if using a different electrolyte according to the redox reaction [36], *n*_0_ is the initial amount of gas moles in the electrolyte reservoir and is related to the initial volume of gas *V*_0_ in the electrolyte reservoir [25] by *P*_0_*V*_0_ = *n*_0_*RT*, and *P*_0_ is the initial value of *P*, i.e., the initial environmental pressure at the target region (e.g., ~117 kPa when considering blood pressure).

The pressures applied at both sides of the flexible membrane can be combined with the pressure differential in the microfluidic channel given by *P*_drug_ − *P*_0_ to derive the pressure *P* from equilibrium in the device as [23]

(5) $$\begin{array}{c} P=f\left(V\right)+P_{0}+\frac{12\mu L\dot{V}}{ab^{3}\left(1-0.63\left(b/a\right)\right)}, \end{array}$$

where the terms on the right-hand side include the resistance to membrane deformation, the initial environmental pressure *P*_0_ at the target region, and the microfluidic resistance that includes the following: *μ* is the dynamic viscosity of the drug, *L* is the length of the microchannels, *a* and *b* are the width and height of the rectangular microchannel, and $\dot{V}$ is the drug flowrate. The microfluidic resistance term $P_{\text{drug}}-P_{0}=12\mu L\dot{V}/\left(ab^{3}\left(1-0.63\left(b/a\right)\right)\right)$ is given for a rectangular cross section with laminar flow and must be modified if using different cross sections; for instance, in the following analysis, it will be simplified to $\sim \left(32\mu L/a^{4}\right)\dot{V}$ for a square (i.e., *a* = *b*) cross section. At the beginning of the drug delivery process (i.e., *t* = 0) before any electrical current flows through the electrodes, the initial value of *P* in Equation (5) is $P=P_{0}+\left(32\mu L/a^{4}\right){\left.\dot{V}\right|}_{t=0}$ which satisfied the presence of an initial amount of gas moles in the electrolyte reservoir *n*_0_. Substituting the pressure *P* and number of moles *n* into the ideal gas law in Equation (4) yields a 1^st^-order ordinary differential equation (ODE) for the drug volume *V* as

(6) $$\begin{array}{c} t=\frac{4F}{3iRT}\left\{\;P_{0}V+f\left(V\right)\left(V+V_{0}\right)+\frac{32\mu L}{a^{4}}\left[\dot{V}\left(V+V_{0}\right)-{\left.\dot{V}\right|}_{t=0}V_{0}\right]\right\}. \end{array}$$

The first-order ODE in Equation (6) is the governing equation for the drug delivery process, and it can be rewritten nondimensionally by normalizing the volume as *V*^∗^ = *V*/*R*_0_^3^, introducing a nondimensional time *t*^∗^ = *t*((3/4)(*RT*/*F*)(*i*/*EhR*_0_^2^)) , and writing the general expression for the function *f*(*V*) nondimensionally as *G*(*V*^∗^) = (*R*_0_/*Eh*)*f*(*V*^∗^*R*_0_^3^) to yield

(7) $$\begin{array}{c} t^{*}=P_{0}^{*}V^{*}+G\left(V^{*}\right)\left(V^{*}+V_{0}^{*}\right)+M^{*}\left[\frac{dV^{*}}{dt^{*}}\left(V^{*}+V_{0}^{*}\right)-{\left.\frac{dV^{*}}{dt^{*}}\right|}_{t^{*}=0}V_{0}^{*}\right] \end{array}$$

that involves three nondimensional parameters, namely, the initial environmental pressure *P*_0_^∗^ = (*R*_0_/*Eh*)*P*_0_, the initial volume *V*_0_^∗^ = (*V*_0_/*R*_0_^3^), and the microfluidic resistance *M*^∗^ = (24*μL*/*a*^4^)(*RT*/*F*)(*R*_0_^2^/*E*^2^*h*^2^)*i* that combine all the dimensional parameters involved in the delivery process into only three nondimensional parameters that can be studied independently to understand how they influence the drug delivery process. In this case, it is important to note that the nondimensional function *G*(*V*^∗^) in Equation (7) is the nonlinear function obtained from FEA as shown in Figure 2(b) because the governing equation is used to obtain the volume temporal profile. The governing equation in Equation (7) can be solved numerically with the initial condition *V*^∗^(*t*^∗^ = 0) = 0.

### 2.3. Analytical Model for Flowrate in Drug Delivery: Slow Variable

In general, the microfluidic resistance *M*^∗^ is small, as compared to the other two nondimensional parameters *P*_0_^∗^ and *V*_0_^∗^, but not zero (*M*^∗^ ≪ 1), such that the perturbation method [37] can be used to solve the governing equation in Equation (7) analytically [25] or the drug delivery time as

(8) $$\begin{array}{c} t^{*}=P_{0}^{*}V^{*}+G\left(V^{*}\right)\left(V^{*}+V_{0}^{*}\right)+M^{*}\left[\frac{V^{*}+V_{0}^{*}}{P_{0}^{*}+G\left(V^{*}\right)+G^{\prime }\left(V^{*}\right)\left(V^{*}+V_{0}^{*}\right)}-\frac{V_{0}^{*}}{P_{0}^{*}+G^{\prime }\left(0\right)V_{0}^{*}}\right], \end{array}$$

which is written explicitly for the total nondimensional delivery time *t*^∗^ that it takes to deliver a nondimensional volume of drug *V*^∗^. The first two terms on the right side of Equation (8) can be regarded as the time required for the flexible membrane to deform and overcome the external environmental pressure and the last term is as the time for the drug to travel through the microfluidic channels where both of these times occur simultaneously; i.e., as the flexible membrane deforms, it pumps the drug through the microchannels. Let *V*^∗^ = *V*_slow_^∗^(*t*^∗^) denote the solution of the above equation, where the subscript “slow” is used to denote a function of the regular (slow) time *t*^∗^ (as opposed to the fast time introduced in the next section). The flowrate of the “slow” variable solution is obtained by taking its derivative with respect to time in Equation (8) as

(9) $$\begin{array}{c} \frac{dV_{\text{slow}}^{*}}{dt^{*}}={\left\{P_{0}^{*}+G\left(V^{*}\right)+G^{\prime }\left(V^{*}\right)\left(V^{*}+V_{0}^{*}\right)+M^{*}\frac{P_{0}^{*}-G^{\prime }\left(V^{*}\right)\left(V^{*}+V_{0}^{*}\right)-G^{\prime \prime }\left(V^{*}\right){\left(V^{*}+V_{0}^{*}\right)}^{2}+G\left(V^{*}\right)}{{\left[P_{0}^{*}+G\left(V^{*}\right)+G^{\prime }\left(V^{*}\right)\left(V^{*}+V_{0}^{*}\right)\right]}^{2}}\right\}}^{-1}. \end{array}$$

It is important to note that Equation (9) gives a nonzero initial flowrate that is equal to

(10) $$\begin{array}{c} {\left.\frac{dV_{\text{slow}}^{*}}{dt^{*}}\right|}_{t^{*}=0}={\left\{P_{0}^{*}+G^{\prime }\left(0\right)V_{0}^{*}+M^{*}\frac{P_{0}^{*}-G^{\prime }\left(0\right)V_{0}^{*}-G^{\prime \prime }\left(0\right){V_{0}^{*}}^{2}}{{\left[P_{0}^{*}+G^{\prime }\left(0\right)V_{0}^{*}\right]}^{2}}\right\}}^{-1}, \end{array}$$

which does not satisfy the zero-flowrate initial condition (*dV*^∗^/*dt*^∗^)|_*t*^∗^=0_ = 0.

### 2.4. Analytical Model for Flowrate in Drug Delivery: Fast Variable

The “slow” variable solution *V*^∗^ = *V*_slow_^∗^(*t*^∗^) presented in the previous section works well when *M*^∗^ is small except at the initial delivery time because it does not satisfy the zero-flowrate initial condition. Since *M*^∗^ appears as the highest order derivative in the “slow” variable solution for the flowrate in Equation (9) and is small, the singular perturbation method can be used to introduce a “fast” variable solution of the form *M*^∗^*V*_fast_^∗^(*η*), where the *η* = *t*^∗^/*M*^∗^ is a “fast” changing variable and the presence of *M*^∗^ ensures a small effect on the volume temporal profile but a large effect initially in the flowrate to satisfy the zero-flowrate initial condition.

The total drug delivery time in Equation (8) then becomes *V*^∗^ = *V*_slow_^∗^(*t*^∗^) + *M*^∗^*V*_fast_^∗^(*η*), where *t*^∗^ is the “slow” variable and *η* = *t*^∗^/*M*^∗^ is a “fast” changing variable that is relevant near the initial time of the delivery process to ensure a zero initial flowrate. Therefore, for a finite *η* and a very small *M*^∗^, the value of *ηM*^∗^ is approximately zero such that *V*_slow_^∗^(*t*^∗^ = 0) = 0, *G*(0) = 0, and (*dV*_slow_^∗^/*dt*^∗^)|_*t*^∗^=0_ is a constant value given from Equation (10) and these assumptions can be used to derive *V*_fast_^∗^ as

(11) $$\begin{array}{c} V_{\text{fast}}^{*}=-\frac{V_{0}^{*}}{P_{0}^{*}+G^{\prime }\left(0\right)V_{0}^{*}}{\left.\frac{dV_{\text{slow}}^{*}}{dt^{*}}\right|}_{t^{*}=0}\langle 1-e^{\left\{-\left[\frac{P_{0}^{*}}{V_{0}^{*}}+G^{\prime }\left(0\right)\right]\frac{t^{*}}{M^{*}}\right\}}\rangle , \end{array}$$

and the derivation details for the “fast” variable are shown in the Supplementary Information (Note 2). The flowrate term of the “fast” variable solution is obtained from Equation (11) by taking a derivative as

(12) $$\begin{array}{c} \frac{dV_{\text{fast}}^{*}}{d\eta^{*}}=-{\left.\frac{dV_{\text{slow}}^{*}}{dt^{*}}\right|}_{t^{*}=0}e^{\left\{-\left[\frac{P_{0}^{*}}{V_{0}^{*}}+G^{\prime }\left(0\right)\right]\eta \right\}}. \end{array}$$

The complete expression for the flowrate is *dV*^∗^/*dt*^∗^ = (*dV*_slow_^∗^/*dt*^∗^) + (*dV*_fast_^∗^/*dη*). For time *t*^∗^ = 0, Equation (12) becomes *dV*_fast_^∗^/*dη*^∗^|(*dV*_fast_^∗^/*dη*^∗^)|_*η*=0_ = −(*dV*_slow_^∗^/*dt*^∗^)|_*t*^∗^=0_ to satisfy the initial condition of zero initial flowrate.

The following terminology is adopted in this section to distinguish the solutions:

- The term “numerical” is used for solutions of the ODE in Equation (7) described in Section 2.2 when the nondimensional function *G*(*V*^∗^) is obtained from FEA using the Marlow hyperelastic model based on the stress-strain behavior of the SIS polymer
- The term “semianalytical slow” is used for the “slow” variable solutions in Equation (9) described in Section 2.3 for the flowrate defined as *dV*^∗^/*dt*^∗^ = *dV*_slow_^∗^/*dt*^∗^ when the nondimensional function *G*(*V*^∗^) is obtained from FEA using the Marlow hyperelastic model based on the stress-strain behavior of the SIS polymer
- The term “semianalytical slow + fast” is used for the solutions in Equations (9) and (12) described in Section 2.4 for the flowrate defined as *dV*^∗^/*dt*^∗^ = (*dV*_slow_^∗^/*dt*^∗^) + (*dV*_fast_^∗^/*dη*) when the nondimensional function *G*(*V*^∗^) is obtained from FEA using the Marlow hyperelastic model based on the stress-strain behavior of the SIS polymer
- The term “analytical slow + fast” is used for the solutions in Equations (9) and (12) described in Section 2.4 for the flowrate defined as *dV*^∗^/*dt*^∗^ = (*dV*_slow_^∗^/*dt*^∗^) + (*dV*_fast_^∗^/*dη*) when the nondimensional function *G*(*V*^∗^) is derived from plate theory for bending-dominated deformation in Equation (2)

**Figure 3:**
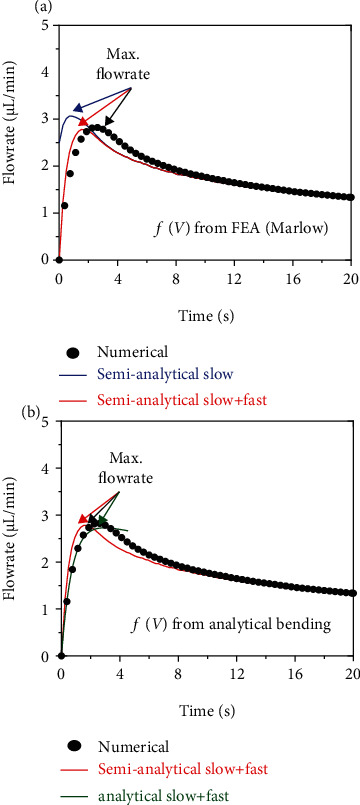
Flowrate temporal profile during drug delivery. (a) Representative example of the flowrate temporal profile obtained from the numerical, semianalytical slow, and semianalytical slow + fast solutions for a bioelectronic device when the function *f*(*V*) is obtained from the finite element analysis (FEA) using the Marlow hyperelastic model. The maximum flowrate is labeled as the peak value of the flowrate temporal profile. (b) Representative example of the flowrate temporal profile showing the analytical slow + fast solution where the function *f*(*V*) is obtained from bending-dominated deformation. The dimensions of the flexible membrane are thickness *h* = 150 *μm* and radius *R*_0_ = 1.20 mm. The electrical current is 0.5 mA, and the cross section of the microchannels is 50 *μ*m. The three nondimensional values are *M*^∗^ = 0.0009, *P*_0_^∗^ = 0.1013, and *V*_0_^∗^ = 0.9162.

The results in Figure 3 show the flowrate temporal profile for a representative bioelectronic device with a SIS flexible membrane previously used for combined drug and light delivery in the mouse brain [10] with the parameters listed in Table 2 and show the numerical, semianalytical, and analytical solutions of the flowrate and its maximum value. Figures 3(a) and 3(b) both show the numerical solution and “semianalytical slow + fast” solution, and they begin with an initial zero flowrate and increase until reaching a peak value labeled as the maximum flowrate and then gradually decrease as the drug delivery process continues. The main difference between the “semianalytical slow” and “semianalytical slow + fast” solutions in Figure 3(a) is at the beginning of the delivery process (i.e., *t* = 0) showing that the “semianalytical slow” solution does not satisfy the zero initial flowrate, but the “semianalytical slow + fast” solution does due to the introduction of the “fast” variable *η* = *t*^∗^/*M*^∗^ which dominates at the beginning of the delivery process. Both semianalytical solutions in Figure 3(a) closely match the numerical solution after the initial time because the function *f*(*V*) is obtained from FEA based on the Marlow hyperelastic model and it considers both the bending and stretching effects of the deformation. Thus, just like in the previous volume temporal profile models [24, 25], modeling the flowrate temporal profile requires excellent agreement between the FEA and analytical function *f*(*V*). However, since the maximum flowrate always occurs at the beginning of the drug delivery process when the bending effects in the flexible membrane are prevalent, the FEA function *f*(*V*) can be replaced by the linear *f*(*V*) function given in Equation (1) for bending-dominated deformation to model the drug delivery process up to the point where the maximum flowrate is reached. For this bioelectronic device geometry specifically (i.e., thickness *h* = 150 *μ*m and radius *R*_0_ = 1.2 mm), the bending effects in the membrane cannot be neglected when controlling the maximum flowrate. Figure 3(b) shows that the “analytical slow + fast” using the in *f*(*V*) given in Equation (1) satisfies the zero initial flowrate condition due to the presence of the “fast” variable and has excellent agreement with the numerical solution up to the point of the maximum flowrate which is the key quantity of focus in this analysis. It is important to note that the bending-dominated deformation *f*(*V*) can only be used up to the time point when the maximum flowrate is reached while the deformation remains small; otherwise, a stretching-dominated *f*(*V*) or the FEA solution *f*(*V*) is necessary. The reason why the bending-dominated solution *f*(*V*) is relevant in this particular case is twofold: (1) the maximum flowrate always occurs near the beginning of the drug delivery process when the deformation is small and therefore bending-dominated and (2) the flexible membrane bending effects depend on the nondimensional ratio *h*/*R*_0_ = 0.125 which is almost five times higher than bioelectronic devices handling larger drug volumes (e.g., 100–1000 *μ*l) that focus on achieving faster drug delivery *h*/*R*_0_ = 0.03, where a larger membrane radius ensures stretching-dominated deformation [25] and the control is on the drug delivery time and volume instead of the flowrate and its maximum value. However, for compact bioelectronics with drug delivery capabilities for use in small animals, the key quantity to control is the magnitude of the maximum flowrate to avoid damaging surrounding fragile tissues resulting from excessively high flowrates, not the volume or temporal profile as shown in our previous work [23–25] where the focus was to obtain the total delivery time and volume.

### 2.5. Analytical Model for Maximum Flowrate

**Figure 4:**
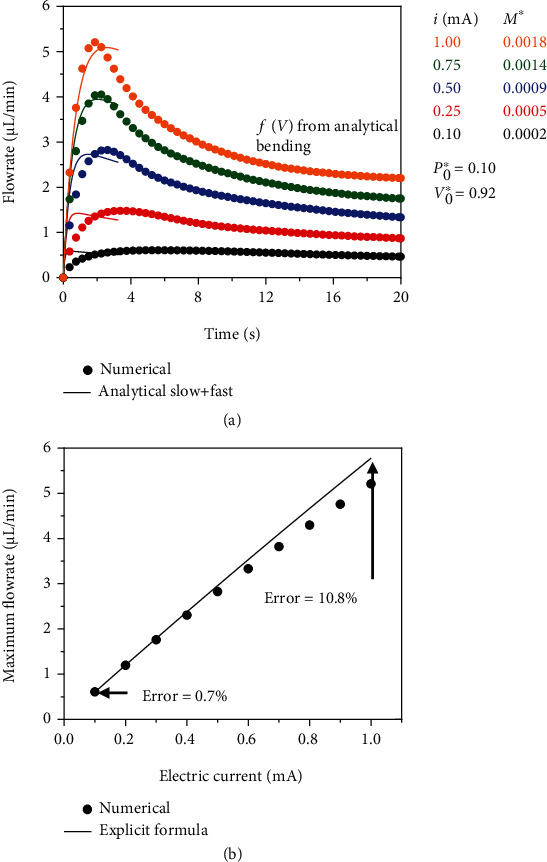
Maximum flowrate. (a) Drug delivery temporal flowrate showing the maximum (peak) flowrate value when the electrical current changes from 0.10 mA to 1.00 mA. (b) Maximum flowrate as a function of the electrical current. The dimensions of the flexible membrane are thickness *h* = 150 *μ*m and radius *R*_0_ = 1.20 mm. The cross section of the microchannels is 50 *μ*m. The two nondimensional values are *P*_0_^∗^ = 0.1013 and *V*_0_^∗^ = 0.9162.

The flowrate temporal profiles in Figure 4(a) show that the maximum flowrate occurs near the beginning of the drug delivery process. Currently, the “analytical slow + fast” solution for the flowrate is divided into the “slow” and “fast” terms given by *dV*^∗^/*dt*^∗^ = (*dV*_slow_^∗^/*dt*^∗^) + (*dV*_slow_^∗^/*dη*), and the exact time when the maximum flowrate occurs can be obtained from *d*^2^*V*^∗^/*dt*^∗2^ = (*d*^2^*V*_slow_^∗^/*dt*^∗^) + (1/*M*^∗^)(*d*^2^*V*_fast_^∗^/(*dη*^2^)) = 0, which, however, is difficult to yield an explicit formula for the exact time when the maximum flowrate occurs. When time is small, such as in the beginning of the delivery process (i.e., *t*^∗^ = 0), the term *d*^2^*V*_slow_^∗^/*dt*^2∗^ can be approximated by the constant (*d*^2^*V*_slow_^∗^/*dt*^∗2^)|_*t*^∗^=0_ because in the “slow” variable solution, the maximum flowrate always occurs near *t*^∗^ = 0, but *η* in the “fast” variable solution is not zero and must be determined. Then, the approximate time when the maximum flowrate occurs can be rewritten as

(13) $$\begin{array}{c} \frac{d^{2}V^{*}}{d{t^{*}}^{2}}={\left.\frac{d^{2}V_{\text{slow}}^{*}}{d{t^{*}}^{2}}\right|}_{t^{*}=0}+\frac{1}{M^{*}}\frac{d^{2}V_{\text{fast}}^{*}}{d\eta^{2}}=0, \end{array}$$

where the first term in the right-hand side of Equation (13) is a constant and the second term is only a function of *η*. This gives an explicit formula for *η* at which the maximum flowrate occurs, and its substitution into the flowrate expression *dV*^∗^/*dt*^∗^ = (*dV*_slow_^∗^/*dt*^∗^)|_*t*^∗^=0_ + (*dV*_fast_^∗^/*dη*) gives an explicit formula for the maximum flowrate as

(14) $$\begin{array}{c} \max\left(\frac{dV^{*}}{dt^{*}}\right)={\left[P_{0}^{*}+G^{\prime }\left(0\right)V_{0}^{*}\right]}^{-1}\left[1-M^{*}\frac{1\;}{{\left[P_{0}^{*}+G^{\prime }\left(0\right)V_{0}^{*}\right]}^{2}}\right], \end{array}$$

where all the derivation details are shown in the Supplementary Information (Note 3). Since the bending effects are relevant at the beginning of the drug delivery process when the maximum flowrate occurs, the expression for *G*′(0) can be derived from Equation (1) as *G*′(0) = (64/3*π*)(*h*^2^/*R*_0_^2^). The explicit nondimensional expression for the maximum flowrate becomes

(15) $$\begin{array}{c} \max\left(\frac{dV^{*}}{dt^{*}}\right)={\left[P_{0}^{*}+\frac{64}{3\pi }\frac{h^{2}}{R_{0}^{2}}V_{0}^{*}\right]}^{-1}\left[1-M^{*}\frac{1\;}{{\left[P_{0}^{*}+\left(64/3\pi \right)\left(h^{2}/R_{0}^{2}\right)V_{0}^{*}\right]}^{2}}\right] \end{array}$$

that depends on the ratio (*h*/*R*_0_)  (due to the bending effects) and the three nondimensional parameters *P*_0_^∗^, *V*_0_^∗^, and *M*^∗^ described in Section 2.3. Typically, in these bioelectronic devices with the representative parameters listed in Table 2, the electrical current can be modulated to control (increase or decrease) the maximum flowrate as shown in Figure 4(a) where the electrical current is changed from 0.10 mA to 1.00 mA which in turn increases the maximum flowrate from 0.60 *μ*l/min to 5.20 *μ*l/min. The corresponding nondimensional parameters for this example are calculated and shown in Figure 4(a) for comparison, where *M*^∗^ increases from 0.0002 to 0.0018 when the electrical current changes and the other two nondimensional parameters *P*_0_^∗^ and *V*_0_^∗^ are fixed since they do not depend on the electrical current.

The “analytical slow + fast” solution in Figure 4(a) shows excellent agreement up to the time when the maximum flowrate is reached; after, the agreement deteriorates for larger current values (e.g., 0.75 and 1.00 mA) due to (1) the magnitude of *M*^∗^ which increases and (2) the differences between the function *f*(*V*) obtained from FEA and bending-dominated deformation in Equation (1). Most notably, the magnitude of the maximum flowrate shows excellent agreement between the numerical and “analytical slow + fast,” which is the key quantity to control during the drug delivery process to avoid damaging fragile surrounding tissues. Figure 4(b) shows the excellent agreement of the value for the maximum flowrate obtained from the explicit analytical formula in Equation (15) with the numerical values computed from the peaks in the flowrate temporal profile. When the electrical current is less than 0.50 mA, the agreement between the numerical and explicit formula is excellent. As the electrical current increases to 1.00 mA, the explicit formula overpredicts the maximum flowrate by ~10.8%, which is still a reasonable agreement, which validates the explicit analytical expression in Equation (15) for the maximum flowrate.

### 2.6. Parametric Study of the Maximum Flowrate

**Figure 5:**
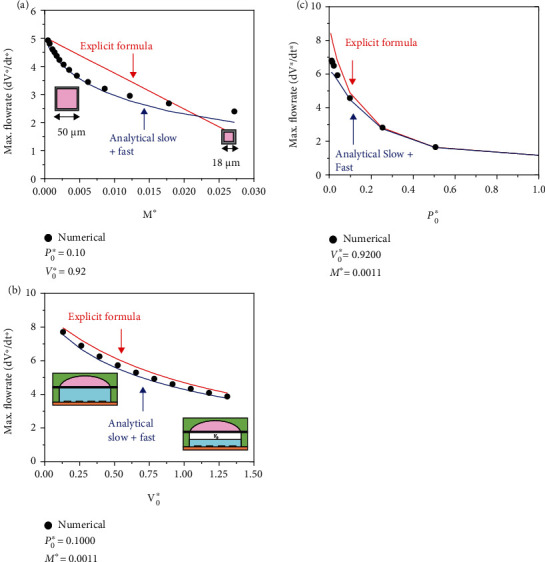
Parametric study of the maximum flowrate. Changes in the maximum flowrate when (a) the microchannel cross section is reduced from 50 *μ*m to 18 *μ*m which increases the nondimensional parameter *M*^∗^, (b) the electrolyte chamber goes from full to partially full (50%) which introduces an initial gas volume via the nondimensional parameter *V*_0_^∗^, and (c) the initial environmental pressure in the tissue/organ changes which affects the nondimensional parameter *P*_0_^∗^. The dimensions of the flexible membrane are thickness *h* = 150 *μ*m  and radius *R*_0_ = 1.20 mm.

The maximum flowrate in Equation (15), like the scaling law in Equation (7), depends on the three nondimensional parameters *P*_0_^∗^, *V*_0_^∗^, and *M*^∗^. Therefore, understanding how the maximum flowrate scales with each of the three nondimensional parameter is important to design and optimize the bioelectronic device geometry and ensure safe and successful drug delivery. To explore the influence of *M*^∗^, the cross-sectional area of the microfluidic channel *a* is reduced from 50 *μ*m to 18 *μ*m which in turn increases *M*^∗^ to 0.027, while the other two nondimensional parameters are fixed to *P*_0_^∗^ = 0.10 and *V*_0_^∗^ = 0.92. This cross-sectional reduction is relevant when targeting smaller areas (or cells) within the tissues to ensure that the drug is being delivered only in a specific region.  Figure 5(a) shows that the maximum nondimensional flowrate decreases approximately nonlinearly with *M*^∗^; this nonlinearity is clearly captured from the flowrate temporal profile peaks in the “analytical slow + fast” model as shown by the excellent agreement with the numerical solution. The explicit formula in Equation (15) has a linear dependence on *M*^∗^ as shown in Figure 5(a) and provides an excellent agreement when *M*^∗^ is small (key assumption when using the perturbation method) and a reasonably well analytical approximation to the nondimensional maximum flowrate when *M*^∗^ increases over the relevant range of microchannel cross sections.

To study the influence of *V*_0_^∗^, which is relevant in the refill ability aspect of the bioelectronic device, the other two nondimensional parameters were fixed to *P*_0_^∗^ = 0.1000 and *M*^∗^ = 0.0011.  Figure 5(b) shows that both the “analytical slow + fast” and the explicit formula agree extremely well with the numerical solution in the range *V*_0_^∗^ = 0 − 1.3, which corresponds to the electrolyte reservoir being completely full *V*_0_^∗^ = 0 and partially full (i.e., 50%) that introduces the presence of an initial gas volume *V*_0_^∗^ = 1.3. Here, the explicit formula slightly overpredicts within ~6% the magnitude of the nondimensional maximum flowrate.

To understand the influence of *P*_0_^∗^, the other two nondimensional parameters are fixed to *V*_0_^∗^ = 0.9200 and *M*^∗^ = 0.0011. Significantly changing the value of *P*_0_^∗^ is difficult as it depends on the initial environmental pressure of the target organ which might vary only by a few kilopascals in humans.  Figure 5(c) shows that the “analytical slow + fast” and the explicit formula agree very well with the numerical solution.

## 3. Discussion

Wireless drug delivery technologies have attracted significant attention for their ability to precisely deliver small drug volumes in a programmable fashion to different target tissues/organs without restricting the animal's ability to move. Designing these drug delivery technologies requires careful consideration of the geometrical, electrical, flexural, fluidic, and environmental parameters to optimize the device layout to ensure safe and complete delivery within the required timeframe based on the flowrate and delivery time. Specifically, controlling the maximum flowrate during the drug delivery process can help to reduce any damage to the surrounding fragile tissues caused by stresses generated from excessively high flowrates. The introduction of a “fast” variable analytical model in Section 2.4 and the explicit formula for the maximum flowrate in Section 2.5 provides a theoretical framework to control the maximum flowrate by changing any of the three nondimensional parameters *P*_0_^∗^, *V*_0_^∗^, and *M*^∗^. Future flexible bioelectronic systems with drug delivery capabilities can be designed by explicitly considering the influence of geometric dimensions, electronics, flexible membrane mechanics, and microfluidic geometries. For example, wireless drug delivery devices in neuroscience experiments are aimed at being small and lightweight such as to not influence the behavior of the small animal during experiments. This design goal of achieving a small and functional device can be achieved by carefully studying the unique combinations of the nondimensional parameters to yield the optimal configuration for the bioelectronic device. After the bioelectronic device is fabricated, only the initial gas volume and electric current can be modified. The initial gas volume can be changed during the refilling process between experiments, and the electric current can be modified by operating at different duty cycles. Restrictions in size and geometric dimensions affect the device radius and available electric current, and these effects can be modeled using the nondimensional parameters to identify if the bioelectronic device is able to reach the desired maximum flowrate range during delivery before fabricating the device. Further, the influence of the flexible membrane mechanics can be explicitly considered to use a block copolymer with Young's modulus that deforms quickly with the applied pressure (i.e., soft) and helps achieve the desired maximum flowrate. Similarly, the influence of the microfluidic channel geometry and dimensions can be considered explicitly such as to not impose excessive fluid resistance that can delay the delivery or cause a blockage inside the device while still targeting drug delivery in specific locations. For example, Figure 5(a) shows that changing the cross section of the microchannel from 50 *μ*m (delivery area 2500 *μ*m^2^) to 18 *μ*m (delivery area 324 *μ*m^2^) will decrease the maximum flowrate during delivery which can be important to consider especially when interested in drug delivery to cells with dimensions in the tens of micrometers or less. The separation of the flowrate solution into a “slow” variable (which is relevant to determine the total delivery time) and a “fast” variable (which is relevant to satisfy the zero-flowrate initial condition at the beginning of the drug delivery process) allows to prioritize which dimensional parameters (and consequently nondimensional parameters) to change depending on the quantity to control, e.g., delivery time or flowrate and its maxima, and most importantly how to change them to increase or decrease the maximum flowrate based on the linear dependence on *M*^∗^ and inverse linear dependence on *P*_0_^∗^ and *V*_0_^∗^ shown in Equation (15) which can be used to control the maximum flowrate when changing the microchannel cross section to target a smaller region in the organs, initial volume of gas during refill and reuse process, and the physiological pressure of the target region organ as shown in Figure 5. For example, the proposed analytical model can be used to design the bioelectronic device to comply with the maximum flowrate applications listed in Table 1 and tailor specific experiments, target locations, drug delivery timeframe, and animal size. The physics of the maximum flowrate are presented as follows: the first term in Equation (15) is the maximum flowrate achieved while the flexible membrane is overcoming the external pressure to deform, and the second term (which is a negative term of the nondimensional parameter *M*^∗^) is the delayed effect caused by the drug traveling through the microchannels. The relevance of the “slow + fast” variable analytical model presented in Section 2.4 in the context of time-sensitive experiments in freely moving animals (1) provides control over the rates of drug delivery which are important in many behavioral neuroscience studies and the total time to deliver the drug and (2) the “fast” variable model allows to determine the time required to reach the maximum flowrate to enable safe wireless pharmacology experiments. The benefits of employing the “analytical slow + fast” variable over the numerical model and FEA to design this emerging class of bioelectronic devices are as follows: (1) the iterative design and optimization process can be done in minutes to properly tune the maximum flowrate by studying only the unique combinations of the nondimensional parameters, subject to practical limits in the fabrication process, rather than each individual dimensional parameter (e.g., electric current, device, and microchannel geometries) and help to study only optimized geometries that need to be characterized experimentally using microparticle tracking velocimetry, a confocal microscope technique used to examine flowrate characteristics in microfluidic devices with drug delivery capabilities. Noting that the “slow + fast” variable analysis presented here is for a bioelectronic device where the bending effects in the polymer flexible membrane are not negligible since the maximum flowrate occurs at the beginning of the delivery process, a similar analysis can be performed for bioelectronic devices mainly experiencing large deformation (i.e., stretching-dominated deformation) to determine the total drug delivery time, flowrate, and its maximum value. Although further experimental testing is required to scale these bioelectronics from small animals to medium and large animals for targeted drug delivery studies, the proposed “slow + fast” variable analytical model for the flowrate and its maxima provides a scalable understanding to control the flowrate via *P*_0_^∗^, *V*_0_^∗^, and *M*^∗^ during the drug delivery process.

## 4. Materials and Methods

### 4.1. FEA of the Flexible Membrane

The commercial software ABAQUS was used to calculate the function *f*(*V*) for a flexible membrane with a thickness *h* = 150 *μ*m and radius *R*_0_ = 1.2 mm. A pressure load of *P* was applied uniformly to the bottom surface of the flexible membrane to deform it into the shape of a spherical cap. The flexible membrane was meshed with 3D stress elements (C3D8H), and the total number of 3D elements used in the model is ~50,000. The displacement and rotational degrees of freedom of the element nodes located at the circumference of the flexible membrane were fixed to zero; the remaining elements were free to deform because of the applied pressure resulting in a spherical cap shape. To calculate the deformed volume of the flexible membrane, the changes in the element volume (EVOL) and vertical displacement (U) were output for 250 evenly spaced time intervals. The hyperelastic material properties of the flexible membrane were defined from the uniaxial stress-strain relationship data shown in Figure 2(a) using the Marlow hyperelastic model to build the strain energy function of the SIS block copolymers.

### 4.2. Numerical Model for Drug Delivery

The numerical solver *ode45* was used in MATLAB to solve the 1^st^-order ODE in Equation (6) with the initial condition *V*(*t* = 0) = 0. The step size was set to 1/10,000^th^ of the total time, and the function *f*(*V*) was obtained from FEA. All the parameters used in the model are listed in Table 2.

## Figures and Tables

**Table 1:** Flowrate range in targeted drug delivery applications.

Application	Flowrate	Units	Reference
Intracerebral injection in mice	<0.1	*μ*l/min	[6]
Murine inner ear drug delivery	0.01–0.1	*μ*l/min	[26]
Drug delivery system for the renal medulla in rats	0.016–0.5	*μ*l/min	[27]
Convection-enhanced delivery to striatum in rats	0.1–5	*μ*l/min	[8]
Intracerebroventricular injection of cells in mice	1	*μ*l/min	[28]
Optofluidic drug delivery system for the peripheral nerves	1.5	*μ*l/min	[11]
Optofluidic drug delivery system for the brain	0.1–2.5	*μ*l/min	[10]
Convection-enhanced delivery in the brain of cats	0.5–4	*μ*l/min	[15]
Focal delivery in the brain	0.03–5	*μ*l/min	[9]
Optofluidic drug delivery system for the brain	5.2	*μ*l/min	[29]
Lymphatic drug delivery system	10–80	*μ*l/min	[30]
Intra-arterial drug delivery in rat brain tumor	17–200	*μ*l/min	[7]
Drug delivery system for transdermal delivery	63–520	*μ*l/min	[31]

**Table 2:** Representative values for electrochemical bioelectronic used in drug delivery.

Parameter	Value	Units
*R*_0_	1.2	mm
*E*	8	MPa
*h*	150	*μ*m
*T*	310 (core body temp)	K
*i*	0.10–1.00	mA
*a*, *b*	18–50	*μ*m
*L*	20	mm
*μ*	0.89	mPa-s

## Data Availability

The data sets generated and supporting the findings of this article are obtainable from the corresponding author upon reasonable request. The authors attest that all data for this study are included in the paper.

## Supplementary Materials

Supplementary Materials Supplementary Note 1: derivation of the upper bound time for bending-dominated deformation. Supplementary Note 2: derivation of the analytical model for drug delivery. Supplementary Note 3: derivation of the analytical maximum flowrate.
