# Supplementary material for: Analytical Modeling of Flowrate and Its Maxima in Electrochemical Bioelectronics with Drug Delivery Capabilities
Source: Research (Wash D C). 2022 Mar 4;2022:9805932. doi: 10.34133/2022/9805932 (PMC8917966; doi:10.34133/2022/9805932)
Supplement: Supplementary Materials — Supplementary Note 1: derivation of the upper bound time for bending-dominated deformation. Supplementary Note 2: derivation of the analytical model for drug delivery. Supplementary Note 3: derivation of the analytical maximum flowrate. [file 9805932.f1.docx]

Supplementary Information

**Analytical Modeling of Flowrate and its Maxima in Electrochemical Bioelectronics with Drug Delivery Capabilities.**

Raudel Avila^1^, Yixin Wu^2,3^, Rinaldo Garziera^4^, John A. Rogers^1,2,3,5,6,7,8^ and Yonggang Huang ^1,2,3,9,^ *

**Affiliations**

^1^Department of Mechanical Engineering, Northwestern University, Evanston, IL 60208, USA.

^2^Department of Materials Science and Engineering, Northwestern University, Evanston, IL 60208, USA.

^3^Querrey Simpson Institute for Bioelectronics, Northwestern University, Evanston, IL 60208, USA.

^4^Dipartimento di Ingegneria ed Architettura, Universita’ di Parma, Italy.

^5^Department of Biomedical Engineering, Northwestern University, Evanston, IL 60208, USA.

^6^Department of Electrical and Computer Engineering, Northwestern University, Evanston, IL 60208, USA.

^7^Department of Chemistry, Northwestern University, Evanston, IL 60208, USA.

^8^Department of Neurological Surgery, Northwestern University, Feinberg School of Medicine, Chicago, IL 60611, USA.

^9^Department of Civil and Environmental Engineering, Northwestern University, Evanston, IL 60208, USA.

*Correspondence should be addressed to Yonggang Huang; [y-huang@northwestern.edu](mailto:y-huang@northwestern.edu)

**Supplementary Note 1: Derivation of the Upper Bound Time for Bending-Dominated Deformation.**

For a membrane of thickness of radius $R_{0}$ and thickness $h$. The bending stiffness is $D=\frac{Eh^{3}}{12(1-v^{2})}$ where $E$ is the young modulus, $v$ is the Poisson ratio ($v=0.5$for incompressible solid). Thus, the relationship between the applied pressure and the volume in the membrane is given by **Equation 1** and copied below as

| $f(V)=\frac{16Eh^{3}}{\pi R_{0}^{6}(1-v^{2})}V$ | (S1) |
| --- | --- |

**Equation S1** is valid when the maximum deflection of the membrane $H$ is less than the thickness $h$ i.e., ($H\ll h$). The upper limit of the bending solution is when $H=h$. Beyond this point, the solution no longer holds. Then $H$ can be written as

| $H=\frac{R_{0}^{4}}{64D}f\left( V \right)$ | (S2) |
| --- | --- |

Simplifying the terms in **Equation S2** yields the following condition

| $H=\frac{3}{\pi R_{0}^{2}}V$ | (S3) |
| --- | --- |

Therefore, to use the bending-dominated solution $h$ and $V$ must satisfy

| $h\gg\frac{3}{\pi R_{0}^{2}}V$ | (S4) |
| --- | --- |
| $V\gg\frac{\pi hR_{0}^{2}}{3}$ | (S5) |

and the bending-dominated solution breaks down at the limit when $V=\frac{h\pi R_{0}^{2}}{3}$ .

Rewriting the upper-limit for the bending-dominated deformation in **Equation S5** non-dimensionally as $V^{*}=\frac{\pi h}{3R_{0}}$, then gives $G\left( V^{*} \right)=\frac{64h^{3}}{9R_{0}^{3}}$ and $G'\left( 0 \right)=\frac{64}{3\pi}\frac{h^{2}}{R_{0}^{2}}$. Substituting these definitions into the expression for the drug delivery time given in **Equation 8** gives the critical time (or upper bound time) for the bending dominated solution as

| $t^{*}=P_{0}^{*}\frac{\pi h}{3R_{0}}+\frac{64h^{3}}{9R_{0}^{3}}\left( \frac{\pi h}{3R_{0}}+V_{0}^{*} \right)+M^{*}\left[ \frac{\frac{\pi h}{3R_{0}}+V_{0}^{*}}{P_{0}^{*}+\frac{64h^{3}}{9R_{0}^{3}}+\frac{64}{3\pi}\frac{h^{2}}{R_{0}^{2}}\left( \frac{\pi h}{3R_{0}}+V_{0}^{*} \right)}-\frac{V_{0}^{*}}{P_{0}^{*}+\frac{64}{3\pi}\frac{h^{2}}{R_{0}^{2}}V_{0}^{*}} \right]$ |
| --- |
| (S6) |

For the flexible bioelectronic devices with electrochemical actuation shown here, the maximum flowrate always occurs before the upper bound time in **Equation S6** meaning that the deformation at the maximum flowrate is bending-dominated.

**Supplementary Note 2: Derivation of the Analytical Model for Drug Delivery**

In bio integrated electronics that rely on electrochemical actuation the drug delivery time can be modeled according to the ideal gas law can, where the resistance of the flexible membrane to deformation is considered via the function $f\left( V \right)$, the initial environmental pressure is accounted for by $P_{0}$, and the influence of the microfluidic resistance is calculated based on the microchannel geometry as $\frac{32\mu L}{a^{4}}\dot{V}$ for a square cross-section to yield a first order ODE for the drug volume $V$ shown in **Equation 6** and copy below as

| $t=\frac{4F}{3iRT}\left\{ P_{0}V+f\left( V \right)\left( V+V_{0} \right)+\frac{32\mu L}{a^{4}}\left[ \dot{V}\left( V+V_{0} \right)-\left. \dot{V} \right\vert_{t=0}V_{0} \right] \right\}$ | (S7) |
| --- | --- |

Alternatively, **Equation S7** can be rewritten non-dimensionally as shown in **Equation 7** and copy below

| $t^{*}=P_{0}^{*}V^{*}+G\left( V^{*} \right)\left( V^{*}+V_{0}^{*} \right)+M^{*}\left[ \frac{dV^{*}}{dt^{*}}\left( V^{*}+V_{0}^{*} \right)-\left. \frac{dV^{*}}{dt^{*}} \right\vert_{t^{*}=0}V_{0}^{*} \right]$ | (S8) |
| --- | --- |

that can be solved by using the perturbation method as shown in **Equation 8** and copy below

| $t^{*}=P_{0}^{*}V^{*}+G\left( V^{*} \right)\left( V^{*}+V_{0}^{*} \right)+M^{*}\left[ \frac{V^{*}+V_{0}^{*}}{P_{0}^{*}+G\left( V^{*} \right)+G'\left( V^{*} \right)\left( V^{*}+V_{0}^{*} \right)}-\frac{V_{0}^{*}}{P_{0}^{*}+G'\left( 0 \right)V_{0}^{*}} \right]$ | (S9) |
| --- | --- |

the analytical solution in **Equation S9** is referred to as the “slow” variable solution and can be rewritten as $V^{*}=V_{slow}^{*}(t^{*})$ and does not satisfy the zero-flowrate initial condition as shown in **Equation 10** because the value of $\left. \frac{dV_{slow}^{*}}{dt^{*}} \right|_{t^{*}=0}$ is a non-zero constant. In the “slow” flowrate solution given in **Equation 9** and copied below

| $\frac{dV_{slow}^{*}}{dt^{*}}=\left\{ P_{0}^{*}+G\left( V^{*} \right)+G'\left( V^{*} \right)\left( V^{*}+V_{0}^{*} \right)+M^{*}\frac{P_{0}^{*}-G'\left( V^{*} \right)\left( V^{*}+V_{0}^{*} \right)-G''\left( V^{*} \right)\left( V^{*}+V_{0}^{*} \right)^{2}+G\left( V^{*} \right)}{\left[ P_{0}^{*}+G\left( V^{*} \right)+G'\left( V^{*} \right)\left( V^{*}+V_{0}^{*} \right) \right]^{2}} \right\}^{-1}$ | (S10) |
| --- | --- |

the $M^{*}$ (which is a small term) appears as the highest order term in the “slow” variable flowrate $\frac{dV_{slow}^{*}}{dt^{*}}$ and the singular perturbation method can be used to introduce a “fast” changing solution of the form ${M^{*}V}_{fast}^{*}(\eta)$ where $\eta=\frac{t^{*}}{M^{*}}$ is “fast” changing variable carefully selected to have a very small effect on the volume and a large effect on the flowrate. The “complete” solution for the volume can be written as $V^{*}=V_{slow}^{*}+{M^{*}V}_{fast}^{*}(\eta)$ where the influence of the ${M^{*}V}_{fast}^{*}(\eta)$ term is very small. The derivative of the “complete” flowrate then becomes $\frac{dV^{*}}{dt^{*}}=\frac{dV_{slow}^{*}}{dt^{*}}+\frac{dV_{fast}^{*}}{d\eta}$ where the $\frac{dV_{fast}^{*}}{d\eta}$ is intended to have a large effect at the beginning of the drug delivery process. The term $V_{fast}^{*}(\eta)$ is derived as follows

The 1^st^ order ODE in **Equation S8** can be rewritten as

| $\left( V^{*}+V_{0}^{*} \right) \left[ M^{*}\frac{dV^{*}}{dt^{*}}+G\left( V^{*} \right) \right]+V^{*}P_{0}^{*}- t^{*}=0$ | (S11) |
| --- | --- |

Substitution of $V^{*}=V_{slow}^{*}+{M^{*}V}_{fast}^{*}(\eta)$ and $\frac{dV^{*}}{dt^{*}}=\frac{dV_{slow}^{*}}{dt^{*}}+\frac{dV_{fast}^{*}}{d\eta}$ into **Equation S11** gives

| $\left( V_{slow}^{*}+{M^{*}V}_{fast}^{*}+V_{0}^{*} \right) \left[ M^{*}\frac{dV_{slow}^{*}}{dt^{*}}+M^{*}\frac{dV_{fast}^{*}}{d\eta}+G\left( V_{slow}^{*}+{M^{*}V}_{fast}^{*} \right) \right]+V_{slow}^{*}P_{0}^{*}+{M^{*}V}_{fast}^{*}P_{0}^{*}- t^{*}=0$ | (S12) |
| --- | --- |

For small $M^{*}$, the Taylor series expansion gives $G\left( V_{slow}^{*}+{M^{*}V}_{fast}^{*} \right)=G\left( V_{slow}^{*} \right)+G'\left( V_{slow}^{*} \right){M^{*}V}_{fast}^{*}$. Substituting $t^{*}$ from the $V_{slow}^{*}$ **Equation S9** into **Equation S12** yields

| $\left( V_{slow}^{*}+V_{0}^{*} \right) \left[ M^{*}\frac{dV_{slow}^{*}}{dt^{*}}+M^{*}\frac{dV_{fast}^{*}}{d\eta}+G\left( V_{slow}^{*} \right)+G'\left( V_{slow}^{*} \right){M^{*}V}_{fast}^{*} \right]+{M^{*}V}_{fast}^{*}G\left( V_{slow}^{*} \right)+{M^{*}V}_{fast}^{*}P_{0}^{*}-G\left( V_{slow}^{*} \right)\left( V_{slow}^{*}+V_{0}^{*} \right)-M^{*}\left[ \frac{V_{slow}^{*}+V_{0}^{*}}{P_{0}^{*}+G\left( V_{slow}^{*} \right)+G'\left( V_{slow}^{*} \right)\left( V_{slow}^{*}+V_{0}^{*} \right)}-\frac{V_{0}^{*}}{P_{0}^{*}+G'\left( 0 \right)V_{0}^{*}} \right]=0$ | (S13) |
| --- | --- |

where only the first order terms of $M^{*}$ are kept. Note that $V_{fast}^{*}$ is a function of $\eta$, not of $t^{*}$, and $t^{*}=\eta M^{*}$. Therefore, for a finite $\eta$ and a very small $M^{*}$, the value of $\eta M^{*}$ is approximately zero such that $V_{slow}^{*}\left( t^{*}=0 \right)=0$, $G\left( 0 \right)=0$, and $\left. \frac{dV_{slow}^{*}}{dt^{*}} \right|_{t^{*}=0}$ is a constant value given from **Equation 10**. **Equation S13** is then simplified to the first order ODE

| $\frac{dV_{fast}^{*}}{d\eta}+\left[ \frac{P_{0}^{*}}{V_{0}^{*}}+G'\left( 0 \right) \right]V_{fast}^{*}+\left. \frac{dV_{slow}^{*}}{dt^{*}} \right\vert_{t^{*}=0}=0$ | (S14) |
| --- | --- |

Its solution is

| $V_{fast}^{*}=-\frac{V_{0}^{*}}{P_{0}^{*}+G'\left( 0 \right)V_{0}^{*}}\left. \frac{dV_{slow}^{*}}{dt^{*}} \right\vert_{t^{*}=0}+De^{\left\{ -\left[ \frac{P_{0}^{*}}{V_{0}^{*}}+G^{'}\left( 0 \right) \right]\eta\right\}}$ | (S15) |
| --- | --- |

Where $D$ is a constant determined from the initial condition $\left. \frac{dV^{*}}{dt^{*}} \right|_{t^{*}=0}=0$, therefore $\left. \frac{dV_{slow}^{*}}{dt^{*}} \right|_{t^{*}=0}+\left. \frac{dV_{fast}^{*}}{d\eta} \right|_{\eta=0}=0$ as $D=\frac{V_{0}^{*}}{P_{0}^{*}+G'\left( 0 \right)V_{0}^{*}}\left. \frac{dV_{slow}^{*}}{dt^{*}} \right|_{t^{*}=0}$. The solution for $V_{fast}^{*}$ then becomes **Equation 11** that is copy below as

| $V_{fast}^{*}=-\frac{V_{0}^{*}}{P_{0}^{*}+G'\left( 0 \right)V_{0}^{*}}\left. \frac{dV_{slow}^{*}}{dt^{*}} \right\vert_{t^{*}=0}\left\langle1-e^{\left\{ -\left[ \frac{P_{0}^{*}}{V_{0}^{*}}+G^{'}\left( 0 \right) \right]\frac{t^{*}}{M^{*}} \right\}} \right\rangle$ | (S16) |
| --- | --- |

It is noted that $G'\left( 0 \right)$ is obtained from plate theory for bending-dominated deformation and defined in **Equation 3** as $\frac{64}{3\pi}\frac{h^{2}}{R_{0}^{2}}$ and can be substituted into **Equation S16** to yield

| $V_{fast}^{*}=-\frac{V_{0}^{*}}{P_{0}^{*}+\frac{64}{3\pi}\frac{h^{2}}{R_{0}^{2}}V_{0}^{*}}\left. \frac{dV_{slow}^{*}}{dt^{*}} \right\vert_{t^{*}=0}\left\langle1-e^{\left\{ -\left[ \frac{P_{0}^{*}}{V_{0}^{*}}+\frac{64}{3\pi}\frac{h^{2}}{R_{0}^{2}} \right]\frac{t^{*}}{M^{*}} \right\}} \right\rangle$ | (S17) |
| --- | --- |

And its derivative for the flowrate is given in **Equation 12** and copy below as.

| $\frac{dV_{fast}^{*}}{d\eta^{*}}=-\left. \frac{dV_{slow}^{*}}{dt^{*}} \right\vert_{t^{*}=0}e^{\left[ -\left( \frac{P_{0}^{*}}{V_{0}^{*}}+\frac{64}{3\pi}\frac{h^{2}}{R_{0}^{2}} \right)\eta\right]}$ | (S18) |
| --- | --- |

**Supplementary Note 3: Derivation of the Analytical Maximum Flowrate**

The flowrate temporal profile has a maximum value (peak) that occurs near the initial time of the drug delivery process. The flowrate temporal profile is given by $\frac{dV^{*}}{dt^{*}}=\frac{dV_{slow}^{*}}{dt^{*}}+\frac{dV_{fast}^{*}}{d\eta}$ where the term $\frac{dV_{fast}^{*}}{d\eta}$ satisfies the zero-flowrate initial condition. The exact time when the maximum flowrate occurs can be determined by taking a derivative of the flowrate as

| $\frac{d^{2}V^{*}}{d{t^{*}}^{2}}=\frac{d^{2}V_{slow}^{*}}{d{t^{*}}^{2}}+\frac{1}{M^{*}}\frac{d^{2}V_{fast}^{*}}{d\eta^{2}}=0$ | (S19) |
| --- | --- |

and solving for the value of $t^{*}$which can be used to determine the value of the maximum flowrate. However, the expression in **Equation S19** for the exact time when the maximum flowrate becomes complex to solve explicitly for the time $t^{*}$. Instead, the following assumption is made based on the physics of the drug delivery process: In the “slow” variable term $\frac{d^{2}V_{slow}^{*}}{d{t^{*}}^{2}}$, the maximum flowrate occurs when $t^{*}=0$, therefore this term becomes a constant that can be rewritten as $\left. \frac{d^{2}V_{slow}^{*}}{d{t^{*}}^{2}} \right|_{t^{*}=0}$. Then, the expression for the approximate time when the maximum flowrate occurs becomes

| $\frac{d^{2}V^{*}}{d{t^{*}}^{2}}=\left. \frac{d^{2}V_{slow}^{*}}{d{t^{*}}^{2}} \right\vert_{t^{*}=0}+\frac{1}{M^{*}}\frac{d^{2}V_{fast}^{*}}{d\eta^{2}}=0$ | (S20) |
| --- | --- |

Now, only the value of $\eta$ when the maximum flowrate occurs is to be determined. The term $\frac{d^{2}V_{fast}^{*}}{d\eta^{2}}$ is obtained by taking a derivative of **Equation S18** as

| $\frac{d^{2}V_{fast}^{*}}{d\eta^{2}}=\left[ \frac{P_{0}^{*}}{V_{0}^{*}}+G^{'}\left( 0 \right) \right]\left. \frac{dV_{slow}^{*}}{dt^{*}} \right\vert_{t^{*}=0}e^{\left\{ -\left[ \frac{P_{0}^{*}}{V_{0}^{*}}+G^{'}\left( 0 \right) \right]\eta\right\}}$ | (S21) |
| --- | --- |

Combining the expression for $\left. \frac{d^{2}V_{slow}^{*}}{d{t^{*}}^{2}} \right|_{t^{*}=0}$ and **Equation S21** the $\eta$ when the maximum flowrate occurs can be solved as

| $e^{\left\{ -\left[ \frac{P_{0}^{*}}{V_{0}^{*}}+G^{'}\left( 0 \right) \right]\eta\right\}}=M^{*}\frac{\left[ 2G'\left( 0 \right)+G''\left( 0 \right)V_{0}^{*} \right]\left( \left. \frac{dV_{slow}^{*}}{dt^{*}} \right\vert_{t^{*}=0} \right)^{2}}{\left[ \frac{P_{0}^{*}}{V_{0}^{*}}+G^{'}\left( 0 \right) \right]}$ | (S22) |
| --- | --- |

where only the first order terms of $M^{*}$ are kept. Since the value of $\eta$ when the maximum flowrate occurs is given by **Equation S22**, the approximate expression for the maximum flowrate can be obtained by substituting into $\frac{dV^{*}}{dt^{*}}=\left. \frac{dV_{slow}^{*}}{dt^{*}} \right|_{t^{*}=0}+\frac{dV_{fast}^{*}}{d\eta}$ as

| ${\max\left( \frac{dV^{*}}{dt^{*}} \right)=\left. \frac{dV_{slow}^{*}}{dt^{*}} \right\vert}_{t^{*}=0}\left[ 1-M^{*}\frac{\left[ 2G'\left( 0 \right)+G''\left( 0 \right)V_{0}^{*} \right] \left( \left. \frac{dV_{slow}^{*}}{dt^{*}} \right\vert_{t^{*}=0} \right)^{2}}{\left[ \frac{P_{0}^{*}}{V_{0}^{*}}+G^{'}\left( 0 \right) \right]} \right]$ | (S23) |
| --- | --- |

The term $\left. \frac{dV_{slow}^{*}}{dt^{*}} \right|_{t^{*}=0}$ given in **Equation 10** and can be rewritten as

| $\left. \frac{dV_{slow}^{*}}{dt^{*}} \right\vert_{t^{*}=0}=\left[ P_{0}^{*}+G'\left( 0 \right)V_{0}^{*} \right]^{-1}\left\{ 1+M^{*}\frac{P_{0}^{*}+G'\left( 0 \right)V_{0}^{*}-2G'\left( 0 \right)V_{0}^{*}-G''\left( 0 \right){V_{0}^{*}}^{2}}{\left[ P_{0}^{*}+G'\left( 0 \right)V_{0}^{*} \right]^{3}} \right\}^{-1}$ | (S24) |
| --- | --- |

And the term $\left( \left. \frac{dV_{slow}^{*}}{dt^{*}} \right|_{t^{*}=0} \right)^{2}$ is obtained by squaring **Equation S24** as

| $\left( \left. \frac{dV_{slow}^{*}}{dt^{*}} \right\vert_{t^{*}=0} \right)^{2}=\left[ P_{0}^{*}+G'\left( 0 \right)V_{0}^{*} \right]^{-2}\left\{ 1+M^{*}\frac{P_{0}^{*}+G'\left( 0 \right)V_{0}^{*}-2G'\left( 0 \right)V_{0}^{*}-G''\left( 0 \right){V_{0}^{*}}^{2}}{\left[ P_{0}^{*}+G'\left( 0 \right)V_{0}^{*} \right]^{3}} \right\}^{-2}$ | (S25) |
| --- | --- |

For small $M^{*}$, the Taylor series expansion of **Equation S24** gives

| $\left. \frac{dV_{slow}^{*}}{dt^{*}} \right\vert_{t^{*}=0}=\left[ P_{0}^{*}+G'\left( 0 \right)V_{0}^{*} \right]^{-1}\left\{ 1-M^{*}\frac{P_{0}^{*}+G'\left( 0 \right)V_{0}^{*}-2G'\left( 0 \right)V_{0}^{*}-G''\left( 0 \right){V_{0}^{*}}^{2}}{\left[ P_{0}^{*}+G'\left( 0 \right)V_{0}^{*} \right]^{3}} \right\}$ | (S26) |
| --- | --- |

and the Taylor series expansion of **Equation 19** gives

| $\left( \left. \frac{dV_{slow}^{*}}{dt^{*}} \right\vert_{t^{*}=0} \right)^{2}=\left[ P_{0}^{*}+G'\left( 0 \right)V_{0}^{*} \right]^{-2}\left\{ 1-{2M}^{*}\frac{P_{0}^{*}+G'\left( 0 \right)V_{0}^{*}-2G'\left( 0 \right)V_{0}^{*}-G''\left( 0 \right){V_{0}^{*}}^{2}}{\left[ P_{0}^{*}+G'\left( 0 \right)V_{0}^{*} \right]^{3}} \right\}$ | (S27) |
| --- | --- |

Replacing the terms in **Equation S23** by **Equation S26** and **Equation S27** and keeping only the first order terms of $M^{*}$ gives **Equation 13** that is copy below

| $\max\left( \frac{dV^{*}}{dt^{*}} \right)=\left[ P_{0}^{*}+G'\left( 0 \right)V_{0}^{*} \right]^{-1}\left[ 1-M^{*}\frac{1}{\left[ P_{0}^{*}+G'\left( 0 \right)V_{0}^{*} \right]^{2}} \right]$ | (S28) |
| --- | --- |

Replacing the $G'\left( 0 \right)=\frac{64}{3\pi}\frac{h^{2}}{R_{0}^{2}}$ gives the approximate analytical formula in **Equation 14** and copy below for the value of the maximum flowrate

| $\max\left( \frac{dV^{*}}{dt^{*}} \right)=\left[ P_{0}^{*}+\frac{64}{3\pi}\frac{h^{2}}{R_{0}^{2}}V_{0}^{*} \right]^{-1}\left[ 1-M^{*}\frac{1}{\left[ P_{0}^{*}+\frac{64}{3\pi}\frac{h^{2}}{R_{0}^{2}}V_{0}^{*} \right]^{2}} \right]$ | (S29) |
| --- | --- |
